# Unexpected Detection of Anti-SARS-CoV-2 Antibodies Before the Declaration of the COVID-19 Pandemic

**DOI:** 10.3389/fmed.2022.923715

**Published:** 2022-07-11

**Authors:** Waleed Mahallawi, Nadir Ibrahim

**Affiliations:** Medical Laboratory Technology Department, College of Applied Medical Sciences, Taibah University, Medina, Saudi Arabia

**Keywords:** COVID-19, archived samples, anti-SARS-CoV-2 IgM/IgG, pre-pandemic, SARS-CoV-2

## Abstract

**Background:**

Limited information is currently available regarding the global incidence of severe acute respiratory syndrome coronavirus 2 (SARS-CoV-2) infections prior to the declaration of the coronavirus disease 2019 (COVID-19) pandemic, which may result in improper conclusions regarding the timing of viral transmission.

**Methods:**

We investigated the presence of specific antibodies against the receptor-binding domain (RBD) of SARS-CoV-2 in archived serum samples that were collected from 478 healthy blood donors and patients in Madinah, Saudi Arabia, between October 2019 and January 2020. Enzyme-linked immunosorbent assay (ELISA) was performed to measure SARS-CoV-2 IgM and IgG antibodies. In addition, rheumatoid factor (RF) and urea dissociation tests were performed in all samples, which showed seropositivity for the SARS-CoV-2 IgM antibody. Additionally, Chemiluminescence immunoassays (CLIA) targeting the RBD of the SARS-CoV-2 spike (S) protein were performed to confirm the seropositivity of the samples.

**Results:**

Overall, 20 (4.18%) serum samples were detected by ELISA to have SARS-CoV-2 IgG or IgM antibodies. Of these, 12 (2.51%) samples were positive for IgM antibody, and 8 (1.67%) were positive for IgG antibody. The 12 samples positive for SARS-CoV-2 IgM antibody were subjected to RF and urea dissociation tests, and all samples were RF-negative. The ELISA results were negative for 7 (58.33%) samples when subjected to urea dissociation prior to ELISA, whereas the other 5 (41.67%) samples remained positive. These 5 samples remained positive for the anti-S RBD IgG antibody in the CLIA. In addition, 3 of the 8 samples with IgG positivity according to the ELISA remained positive in the CLIA. After reviewing their data, we discovered that the 8 CLIA-confirmed positive samples were obtained from returned travellers who had visited China during the 4-week period immediately preceding blood donation.

**Conclusion:**

In conclusion, we found evidence to support the early circulation of SARS-CoV-2 among persons who visited China a few months prior to the pandemic declaration. These results can be used to better define the spread of SARS-CoV-2 infections before the COVID-19 pandemic declaration. The detection of SARS-CoV-2 antibodies in individuals before the pandemic was declared in China could rewrite the pre-pandemic timeline.

## Introduction

By the end of December 2019, the novel severe acute respiratory syndrome coronavirus 2 (SARS-CoV-2) virus was identified as the underlying cause of coronavirus disease 2019 (COVID-19), which was first detected in China and rapidly spread worldwide (1). The first case of laboratory-confirmed COVID-19 in the Kingdom of Saudi Arabia was recorded on March 2, 2020. Since then, infections have continued to increase on a daily basis (2). Serologic tests represent a useful tool for assessing the prevalence of SARS-CoV-2 antibodies, which can be used to track viral infection and evaluate its frequency among the population (3). Specific SARS-CoV-2-targeted antibodies have been identified, providing a convenient method for both the clinical surveillance of public health and the monitoring of infected patients (4). However, it has been reported that significant RF reactivity interferes with tested antibodies when immunoassays such as ELISA performed. Additionally, RF has been found to be linked with higher false positive results used in clinical settings as well as researches. (5). Performing population-wide serosurveys for SARS-CoV-2 antibodies can allow for the precise evaluation of the infection rate and can also be used to detect asymptomatic cases (6, 7).

Differences in the humoral immune responses observed among hospitalized patients have revealed a relationship between disease presentation and the immune response mounted to combat the virus (8). Analyses of the antiviral antibody responses in patients diagnosed with COVID-19 indicate that the seroconversion of SARS-CoV-2-targeted IgM and IgG antibodies can be detected within 13 days of symptom onset in nearly all patients with COVID-19 (9).

Abundant information is currently available describing the spread of SARS-CoV-2; however, inadequate evidence has been presented regarding the early spread of the virus leading to the first laboratory-confirmed case. Several studies have been conducted globally to investigate available evidence to help trace the exact or approximate timing of SARS-CoV-2 spread and the viral origins. For example, a recent study reported the evaluation of stored samples obtained from Vietnamese children and adults collected during a pre-pandemic period in Vietnam (2015–2019), which found no evidence of antibodies targeting the SARS-CoV-2 nucleocapsid and spike (S) proteins antibodies in pre-pandemic samples (10). Another study conducted in Italy reported the unexpected early circulation of SARS-CoV-2 among asymptomatic persons in Italy 3 months prior to the detection of the first recognized case, allowing for the more precise mapping of early infections and the spread of the COVID-19 pandemic (11).

Our study investigated the presence of SARS-CoV-2 antibodies in archived serum samples obtained during studies conducted prior to the declaration of the COVID-19 pandemic in Saudi Arabia.

## Materials and Methods

### Serum Samples and Controls

This study examined 478 archived, stored serum samples obtained from blood bank donors and patients in Madinah, Saudi Arabia, during serosurvey projects conducted between October 2019 and January 2020 and stored at −80°C. All blood donors were required to be healthy and disease-free at the time of blood donation. Two serum samples were collected at Madinah General Hospital in February 2022 from symptomatic COVID-19-infected patients <14 days after confirmation of SARS-CoV-2 infection by real-time reverse-transcriptase-polymerase chain reaction (rRT-PCR) and stored at −80°C for use as positive controls. All the positive samples in the current study were from individuals who had visited China prior to the pandemic.

Signed consent forms were obtained from all participants and patients included in this study. Ethical approval to conduct this study was acquired from the Research Ethics Committee of the Institutional Review Board, General Directorate of Health Affairs in Madinah (IRB no: H-03-M-084).

### Serological Assays

#### SARS-CoV-2 IgM and IgG Enzyme-Linked Immunosorbent Assay

Commercially available SARS-CoV-2 Virus IgM and IgG Antibody Detection enzyme-linked immunosorbent assay (ELISA) kits (BGI Europe, Copenhagen, Denmark) were used to detect the presence of SARS-CoV-2 IgM and IgG antibodies in human serum, according to the manufacturer's instructions. The test specificity for IgM antibody detection is 96.76%, the specificity for IgG antibody detection is >98.38%, and the sensitivity for total IgM and IgG detection is >98.71% (https://www.bgi.com/global/molecular-genetics/COVID-19-antibody-detection-kit-elisa/, accessed on March 21, 2022). The results were interpreted as follows: an optical density (OD) greater than the cutoff value was categorized as positive; an OD below the cutoff value was categorized as negative. All washing and reading steps were performed using a semi-automated ELISA washer and reader (Biotek, Winooski, US).

#### Rheumatoid Factor Test

It has been reported that significant RF reactivity interferes with tested antibodies when immunoassays such as ELISA performed. Additionally, RF has been found to be linked with higher false positive result when used in clinical settings as well as researches. To assess the presence of possible interference due to rheumatoid factor (RF) in the SARS-CoV-2 Virus IgM ELISA, sera that tested positive for SARS-CoV-2 IgM were subjected to a semi-quantitative RF latex agglutination slide test (HumaTex RF, Human Gesellschaft fur Biochemica und Diagnostica mbH, Wiesbaden, Germany, CN: 40050), according to the manufacturer's instructions. Test performance characteristics can be found at http://www.human-de.com/data/gb/vr/lx-rf.pdf **(**accessed on March 21, 2022).

#### Urea Dissociation Test

The urea dissociation test was conducted on samples that tested positive for SARS-CoV-2 IgM by ELISA to assess the reliability of positive SARS-CoV-2 IgM results. The results of affinity index (AI) analyses are expressed as the ratios of the OD values obtained for the 4 mol/L concentration of dissociated urea to the OD values obtained from serum samples. The AI threshold value was established as the median value between the highest AI value obtained for all tested serum samples without outliers and the lowest AI value obtained for the rRT-PCR–positive control samples. Sera with AI values greater than or equal to the AI threshold were defined as positive, whereas sera with AI values less than the AI threshold were defined as negative (12).

#### SARS-CoV-2 IgG Chemiluminescence Immunoassays

To confirm positive results detected using the SARS-CoV-2 IgM and IgG ELISA (13), the Elecsys AntiSARS-CoV-2 S immunoassay (La Roche Ltd, Basel, Switzerland), a Chemiluminescence immunoassay (CLIA), was used to provide *in vitro* quantification of antibodies targeting the SARS-CoV-2 S protein receptor-binding domain (RBD). The assay utilizes the RBD of recombinant SARS-CoV-2 S protein in a double-sandwich assay format, with a clinical specificity of >99.98% (https://assets.publishing.service.gov.uk/government/uploads/system/uploads/attachment_data/file/989460/Evaluation_of_Roche_Elecsys_anti_SARS_CoV_2_S_assay_PHE.pdf). The Roche cobas e411 immunoassay analyzer was used according to the manufacturer's instructions. The results are reported as the concentration of anti-SARS-CoV-2 S antibodies detected in each sample (in U/mL), with values <0.8 U/mL categorized as negative and values ≥0.8 U/mL categorized as positive (Roche Diagnostics GmbH. 2022. Elecsys^®^ Anti-SARS-CoV-2 S Assay. Available from: https://diagnostics.roche.com/global/en/products/params/elecsys-anti-SARS-CoV-2.html. Accessed on March 21, 2022). Concentrations in U/mL are directly equivalent to binding antibody units (BAU)/mL defined in the first World Health Organization International Standard for anti-SARS-CoV-2 immunoglobulin detection (NIBSC code 20/136), and results in U/mL may be directly compared to values reported in BAU/mL (14).

### Statistical Analysis

Qualitative variables are presented as the frequency and percentage, whereas quantitative variables are presented as the mean ± standard deviation. Data were analyzed using the Statistical Package for Social Sciences (IBM SPSS Statistics version 25; Armonk, NY, USA).

## Results

### Demographic Characteristics of the Study Population

The demographic characteristics for the study population are shown in Table 1. Of the 478 samples, 301 (62.97%) were obtained from men, 367 (76.78%) were obtained from Saudis, and the population had a mean age of 32.01 ± 10.12 years (range: 18–66 years).

**Table 1:** Demographic characteristics of study population (*n* = 478).

**Parameter**
Sex	Male [*n* (%)]	301 (62.97)
	Female	177 (37.03)
Nationality	Saudi [*n* (%)]	367 (76.78)
	Non-Saudi	111 (23.22)
Age	Age range	18–66
	Age mean (mean ± SD)	32.01 ± 10.12

### Serological Assays for the Detection and Confirmation of SARS-CoV-2 Antibodies

In Table 2, we describe the results of serological assays used to detect and confirm the presence of SARS-CoV-2 antibodies in the study samples. Overall, 20 (4.18%) serum samples were positive for the presence of SARS-CoV-2 IgM or IgG antibodies by ELISA. IgM antibodies were detected in 12 (2.51%) patients, IgG antibodies were found in 8 (1.67%) patients, and both IgM and IgG antibodies were found in only 1 (0.21%) patient. The rRT-PCR–positive control samples were positive for SARS-CoV-2 IgM antibody.

**Table 2:** Serological assays for detection and confirmation of SARS-CoV-2 antibodies in the study samples.

**Samples**	**ELISA**		**RF (IU/mL)**	**ELISA after urea dissociation**	**CLIA (U/mL)**	
	**IgM**	**IgG**			
Sample1	–	+	<12	ND	0.165	–
Sample 2	–	+	<12	ND	0.173	–
Sample 3	–	+	<12	ND	10.071	**+**
Sample 4	–	+	<12	ND	12.899	**+**
Sample 5	–	+	<12	ND	9.045	**+**
Sample 6	–	+	<12	ND	0.103	–
Sample 7	+	+	384	–	0.270	–
Sample 8	–	+	<12	ND	0.146	–
Sample 9	+	–	<12	+	3.118	**+**
Sample 10	+	–	<12	+	2.438	**+**
Sample 11	+	–	<12	–	0.113	–
Sample 12	+	–	<12	–	0.312	–
Sample 13	+	–	<12	–	0.421	–
Sample 14	+	–	<12	+	2.990	**+**
Sample 15	+	–	<12	–	0.395	–
Sample 16	+	–	<12	–	0.521	–
Sample 17	+	–	<12	–	0.234	–
Sample 18	+	–	<12	+	3.806	**+**
Sample 19	+	–	<12	–	0.341	–
Sample 20	+	–	<12	+	4.036	**+**
Positive controls (*n =* 2)	+	ND	ND	+	ND

*ND, not done. (–), negative and (+), positive*.

The 13 samples identified as positive for SARS-CoV-2 IgM antibody by ELISA were subjected to RF and urea dissociation tests. Only one sample tested positive for RF at a concentration of 384 IU/mL. Remarkably, this sample tested positive for both IgM and IgG using the SARS-CoV-2 IgM antibody detection ELISA; however, when the sample was subjected to the urea dissociation test at a concentration of 4 mol/L urea using an AI threshold value of 0.835, the sample was categorized as negative. Additionally, when the sample was subjected to further testing for SARS-CoV-2 antibodies using Elecsys Anti-SARS-CoV-2 S immunoassay, the result was negative for antibodies against the SARS-CoV-2 S protein RBD. The remaining 12 SARS-CoV-2 IgM antibody-positive samples were RF-negative. When the urea dissociation test was performed on these samples and the 2 rRT-PCR-positive controls, the results were negative for 7 samples and remained positive for 5 samples and the rRT-PCR-positive controls.

Notably, antibodies against the SARS-CoV-2 S protein RBD were detected in all 5 samples that remained positive following urea treatment. After reviewing the data for these 5 patients, we discovered that these samples were obtained from non-Saudi foreign students who had traveled to China in the weeks prior to blood donation.

SARS-CoV-2 IgG-positive samples identified by ELISA were subjected to further testing for antibodies against the SARS-CoV-2 S protein RBD using the Elecsys Anti-SARS-CoV-2 S immunoassay. Only 3 samples remained positive following the CLIA.

## Discussion

Scientists continue to seek new information to better understand the earliest periods of the pandemic prior to the global spread of the infection. Various reports have offered additional proof to support the hypothesis that SARS-CoV-2 was initially contracted from animals by the humans who raised, butchered, or bought them. However, currently available information has not yet determined the precise course of events (15–17).

By contrast, a study concerning the origins of the virus indicated that viral origin inquiries commonly take several years and reported that Chinese researchers have already conducted a number of relevant studies (18–20).

In our study, any evidence that supports or opposes published data concerning the viral origins or data that has been withheld from authorities will not be discussed. Our only goal is to disseminate our findings to the scientific community.

We assume that many factors may confound the determination of the exact timing of the pandemic onset and viral spread, such as the under-reporting of SARS-CoV-2 cases. Additionally, a major factor that may have contributed to the difficulties tracing SARS-CoV-2 was the surge of a novel virus commonly associated with asymptomatic cases, which resulted in the spread of the virus among populations without diagnosis. Reports have indicated that at least 50% of transmissions were likely due to asymptomatic individuals (21).

Although useful, serological analyses such as those used in our study to detect the SARS-CoV-2 virus are not error-free, therefore, we applied several assays to verify our findings. To exclude the possibility of RF interference in the SARS-CoV-2 IgM ELISA, we tested our samples for the presence of RF. A majority of the certified serological tests were found to be sensitive to interfering antibodies, such as RF, which are present in the serum of patients who suffer from chronic inflammatory diseases (22).

Our results suggest that the presence of RF IgM may result in false-positive reactivity in the SARS-CoV-2 IgM ELISA (12). Only one sample among the identified IgM-positive samples was also RF-positive. When we performed the ELISA after a urea dissociation step, only 5 of the 12 initially identified positive samples remained positive, indicating that the remaining 7 samples were false positives. We also confirmed our ELISA results by CLIA using the S protein RBD.

One informative study in China verified that the first zoonotic spread of the virus was estimated to have occurred in late November/early December of 2019 and no earlier than the start of November 2019 (23). Thus, the potential spread of the virus among humans at that time is also possible, particularly among asymptomatic individuals. The timeline regarding the emergence of the virus is currently poorly defined; therefore, we assume that the positive samples identified in our study are likely due to sporadic infections among individuals that were not recognized by the government at the time.

Our results detecting anti-SARS-CoV-2 antibodies among individuals who had recently visited China suggest that the virus was present and circulating before the declaration of a pandemic. The blood bank in Madinah performs donation campaigns each year to supply hospitals with blood and blood products for use in patients for medical reasons. In our study, we discovered that the 8 CLIA-confirmed positive samples were obtained from returned travelers who had been in China 2–4 weeks after November 2019. Therefore, the chances that these individuals were infected while in China are very high. However, when they presented for blood donation, they appeared to be healthy and were deemed to be eligible for donation, with no reports of any unusual symptoms, indicating that they experienced asymptomatic infections. Recent data from China indicate that the vast majority of coronavirus infections do not lead to the development of symptoms (24). This report supports our hypothesis that asymptomatic infections are a likely factor contributing to the spread of COVID-19 (25).

Seroconversion following infection varies, ranging from 50% at 11 days after infection to 100% at 39 days post-infection (8, 26). Asymptomatic individuals appear to present with lower levels of seroconversion and antibody persistence (27); however, additional research in larger cohort investigations remains necessary to confirm this observation (28). In our study, we found that 5 samples were IgG-negative and IgM-positive by ELISA, whereas all 5 of these samples were determined to be IgG-positive by CLIA. The seroconversion for IgG and IgM may occur either simultaneously or consecutively, and both IgG and IgM levels plateau within 6 days after seroconversion (9).

In conclusion, we provide evidence to support the unexpected early circulation of SARS-CoV-2 among persons who had visited China a few months prior to the pandemic declaration. These results support the emergence and spread of SARS-CoV-2 before the COVID-19 pandemic declaration. The detection of SARS-CoV-2 antibodies in individuals prior to the reported pandemic eruption in China could rewrite the currently accepted timeline of the pandemic.

Finally, we recommend that scientists in other countries consider analyzing and reporting the results of archived pre-pandemic samples to contribute to clarifying the timeline of the emergence and spread of SARS-CoV-2.

## Data Availability Statement

The raw data supporting the conclusion of this article will be made available by the authors, without undue reservation. Requests to access these datasets should be directed to wmahallawi@gmail.com.

## Ethics Statement

The studies involving human participants were reviewed and approved by Institutional Review Board, General Directorate of Health Affairs in Madinah (IRB No: H-03-M-084). The patients/participants provided their written informed consent to participate in this study.

## Author Contributions

All authors listed have made a substantial, direct, and intellectual contribution to the work and approved it for publication.

## Funding

The authors extend their appreciation to Taibah University, represented by the Deanship of Scientific Research, for funding this project (No. RC-442/4).

## Conflict of Interest

The authors declare that the research was conducted in the absence of any commercial or financial relationships that could be construed as a potential conflict of interest.

## Publisher's Note

All claims expressed in this article are solely those of the authors and do not necessarily represent those of their affiliated organizations, or those of the publisher, the editors and the reviewers. Any product that may be evaluated in this article, or claim that may be made by its manufacturer, is not guaranteed or endorsed by the publisher.
